# Prescriptions

**Published:** 1895-08

**Authors:** 


PAINLESS TOOTH EXTRACTION.

According to the *Medical Standard*, the following has been found of value by many dentists:

℞. Ol. gaultheriæ............ ʒij.

Chloroform............. ʒj.

Ether. sulph.............. ʒj.

Chloralis................. ʒij.

Ol. caryophyllus.... ..... ʒiv.

Alcohol.... ........... ʒxij.

M.—Sig.: Apply with cotton pressed on each side of tooth.

UREMIA.

Dr. L. D. Kastenbine highly recommends:

℞. Tinct. ferri chloridi...... ℥ss.
Spiritus etheris nitrosi... ℥i.
Liq. ammon. acetatis..... ℥i.
Aquæ dest................ ℥iiss.

M. et fiat mistura. Sig.: Tablespoonful in a wineglass of water.—*Louisville Med. Monthly*.

ACUTE BRONCHITIS.

Dr. Eshner often prescribes of the following combination, a teaspoonful every three hours:

℞. Ammonium chlorid...... ʒiii.

Comp. licorice mixture...fl ℥iss.

Camphorated tinc. opium fl ℥iss.

M.

For the "tobacco-heart," in elderly people, give:

℞. Strychnin, arsenate....... gr. j.
Quinin. arsenate......... gr. ij.
Salicin....... .......... ʒj.

M. –Make 60 powders.

Sig.: Give 1 powder after each meal, or ½ powder every three hours, for six doses, dry on the tongue.

This not only tones up the heart, but destroys the appetite for tobacco.—*Philadelphia Polyclinic.*

TAPEWORM.

℞. Oleores. aspidii (filix mas).mvij.

Ol. erigeron..............mj.

Chloral hydrat........ ..gr. iij.

Ol. ricini....... .........mviij.

Ol. tiglii...........m1-20—1-16.

M.—Sig.: Take at one dose. One, two, or three capsules may be required.—*Prescription.*

SWEATING FEET.

By Professor Kaposi:

℞. Powdered talc, gms. 40. (℥jss.)

Subnitrate of bismuth, gms. 45. (℥j¾.)

Permanganate of pot., gms. 3. (grs. xiv.)

Salicylate of soda, gms. 2. (grs. xxx.)

Dust into the shoes every morning.

FISSURES OF THE HANDS.

℞. Menthol... ......... ....gr. xij.

Salol............... ....gr. xxiv.

Ol. olivæ...... ......gr. xxiv.

Lanolin........ ..... ℥iss.

M. ft. ungt.—*La Medecine Moderne.*

OINTMENT

For local inflammatory condition of the skin:

℞. Tinct. opii.............ʒi.

Acid. carbol...........gtt. iii.

Ol amygdal dulc........ʒiii.

Adip. lanæ..... ...... ℥ij.

M.—Ft. ung.

—DR GOTTHEIL

